# Nalbuphine alleviates inflammation by down-regulating NF-κB in an acute inflammatory visceral pain rat model

**DOI:** 10.1186/s40360-022-00573-7

**Published:** 2022-06-01

**Authors:** Dijiao Ruan, Yuanyuan Wang, Sisi Li, Chao Zhang, Wenwen Zheng, Cong Yu

**Affiliations:** 1 Department of Anesthesiology, Stomatological Hospital of Chongqing Medical University, 426 Songs North Road, Yubei District, Chongqing, China; 2 Chongqing Key Laboratory of Oral Diseases and Biomedical Sciences, Chongqing, China; 3 Chongqing Municipal Key Laboratory of Oral Biomedical Engineering of Higher Education, Chongqing, China

**Keywords:** Nalbuphine, NF-κB, Inflammation, Visceral pain

## Abstract

**Introduction:**

Nalbuphine can relieve patients’ inflammation response after surgery compared to other opioid drugs. However, its molecular mechanism has not been clear. Activation of NF-κB signaling pathway under oxidative stress and inflammation can maintain pain escalation.

**Methods:**

We firstly investigated the effect of nalbuphine on writhing test and mechanical allodynia using a rat model of inflammatory visceral pain (acetic acid (AA) administrated). Cytokines (including tumor necrosis factor (TNF)-α, Interleukin (IL)-1β, IL-2, and IL-6 in plasma were tested with ELISA technology. Expression levels of TNF-α, IκBα and p-NF-κB p65 at the spinal cord (L3–5) were measured by western blot or RT-qPCR.

**Results:**

We found that the paw withdrawal threshold (PWT) values of rats were reduced in the model group, while the numbers of writhing, levels of IL-1β, IL-2, IL-6, and TNF-α in plasma, and p-NF-κB protein and its gene expressions in the lumbar spinal cord were up-regulated. Subcutaneously injection of nalbuphine (10 μg/kg) or PDTC (NF-κB inhibitor) attenuated acetic acid-induced inflammatory pain, and this was associated with reversal of up-regulated IL-1β, IL-2, IL-6, and TNF-α in both plasma and spinal cord. Furthermore, acetic acid increased p-NF-κB and TNF-α protein levels in the white matter of the spinal cord, which was attenuated by nalbuphine. These results suggested that nalbuphine can significantly ameliorate inflammatory pain via modulating the expression of NF-κB p65 as well as inflammation factors level in the spinal cord.

**Conclusion:**

In conclusion, nalbuphine inhibits inflammation through down-regulating NF-κB pathway at the spinal cord in a rat model of inflammatory visceral pain.

**Supplementary Information:**

The online version contains supplementary material available at 10.1186/s40360-022-00573-7.

## Introduction

Acute or chronic visceral pain is one of the most commondiseases like pancreatitis, enteritis perihepatitis in our life [1–3]. Approximately 10% of chronic visceral pain is transmitted from acute postoperative pain that is difficult to control [4]. Opioid drugs play an important role in controlling acute pain during most surgical procedures (e.g., gallbladder, gastrointestinal, and gynecological surgery) [5–8].

Compared with μ-opioid receptor agonists such as remifentanil, sufentanil, and fentanyl, nalbuphine is a kappa-opioid receptor agonist and μ-opioid receptor antagonist with fewer side effects and a higher safety profile [9, 10]. Nalbuphine is structurally similar to naloxone, mainly used in clinical practice for intraoperative and postoperative analgesia [11, 12]. Some researchers reported that nalbuphine is primarily acted on kappa receptors to produce analgesia, which can be reversed by naloxone dose-dependently; when acting on μ-receptors, respiratory depression is less than morphine due to the capping effect [13, 14]. There are some studies reported that kappa-opioid drugs are involved in regulating NF-κB signaling in rats to exert a protective effect after myocardial ischemic and suppressed breast cancer stemlike properties [15, 16]. In addition, there are some studies have shown that nalbuphine can inhibit the inflammatory response in patients to help them recover and reduce the length of hospital stay perioperatively. It can reduce the release of proinflammatory cytokines interleukin IL-6, tumor necrosis factor TNF-α, and IL-1β in plasma [17–20]. Moreover, evidence showed that inflammation plays a vital role in visceral pain, it also has a significant impact on short-and long-term postoperative clinical outcomes [21].

Nuclear factor kappa-light-chain-enhancer of activated B cells (NF-κB) is reported to play a critical role in inflammatory degenerative processes [22, 23]. NF-κB-mediated inflammatory responses can lead to progressive extracellular matrix damage [24]. Pain is often accompanied by the activation of various inflammatory cytokines such as IL-1β, IL-2, TNF-α, and NF-κB pathways. In addition, many studies have reported on the role of NF-κB and its downstream pro-inflammatory cytokines in mechanisms underlying inflammatory and neuropathic pain processes [25, 26]. NF-κB is mostly present in the form of a p65-p50 dimer and is associated with inhibitor IkB binds in an inactive state [27]. Studies have shown that the expression of NF-κB p65 is significantly enhanced in various inflammatory models [28–31].

In the preliminary study, we have found that nalbuphine can reduce plasma levels of pro-inflammatory factors such as IL-6 and TNF-α in patients 24 h and 48 h after orthognathic surgery compared with sufentanil [32]. Simultaneously, postoperative nausea, vomiting, and other postoperative complications or anesthesia-related adverse reactions were lower in the nalbuphine group. However, the targets through which nalbuphine exerts its anti-inflammatory and analgesic effects have not been determined.

NF-κB as a key factor in the inflammatory response, there was no report that whether nalbuphine can inhibit the NF-κB signaling pathway in visceral pain rats. Thus, we aimed to test the effect of nalbuphine in a model of inflammatory visceral pain to further clarify the relationship between NF-κB and nalbuphine at the spinal cord in the rat.

## Materials and methods

### Animals and ethics

All methods were carried out in accordance with relevant guidelines and regulations. Specifically, all procedures in our study were carried out in accordance with the ARRIVE guidelines and were approved by the Ethics Committee of the College of Stomatology, Chongqing Medical University (Project No. CQHS-REC-2021 (LSNo. 011)). Forty-eight SPF male Sprague-Dawley rats (8 weeks; 230–260 g) were purchased from Hunan SJA Laboratory Animals (Source, SCXK-2019-0004; Number of qualitative qualification, 430,727,200,101,046,971; Hunan, China). They were housed in the Animal Care Facility within the Chongqing Key Laboratory of Oral Diseases and Biomedicine (Number permit, SYXK-2019-0004) under a 12 h light-dark cycle (lights-on at 7:50 am; lights-off at 7:50 pm). Rats had free access to water and food in a room at 22–25 °C and 50–60% humidity. All rats were fasted but not water-deprived 12 h before experimentation. Rats were divided randomly into four groups named as follows: control, AA, AA+ nalbuphine, and AA+PDTC, with 12 rats in each group. Every rat was experienced writhing test and PWT measurement. A total of 32 heart blood samples were collected from 48 rats, with 8 rats in each group. After blood was collected, the spinal cord of the rats was exposed for WB and PCR. A spinal cord specimen from a rat only was used for one experimental technique. Then, the remaining 16 rats were treated with cardiac perfusion using paraformaldehyde and their spinals cord were exposed for immunohistochemistry. Efforts were made to minimize the number of animals used and the stress they experienced.

### Drugs and agents

Nalbuphine hydrochloride was purchased from Yichang Human Well Pharmaceuticals (State Food and Drug Administration approval number: 989RG; batch number: H20054171; Hunan, China). Antibodies were obtained from primary antibodies against the anti-GAPDH (2118S, CST, USA), anti-TNF-α (GB11188S, Servicebio), anti-IL-6 (ab259341, Abcam, USA), anti-IL-1β (12703S, CST, USA), anti-IκBα (4812 s, CST, USA), anti-phosphorylated (p)-NF-kB p65 (ser536) (310,013, Zen Bioscience). Pyrrolidine dithiocarbamate (PDTC, S1808, Beyotime).

### Inflammatory visceral pain

The effect of nalbuphine on acute inflammatory visceral pain was assessed in a rat model induced by acetic acid. This method was modified from the research description of Satyanarayana PSV et al. [33]. Briefly, it was induced with an intraperitoneal injection of 1 ml/100 g of 1% acetic acid solution into the rats’ peritoneal under isoflurane anesthesia. First, PDTC was administered intraperitoneally to the rats in the PDTC group at 120 mg/kg and then molded with acetic acid 30 min later [34]. Then, under isoflurane anesthesia, rats in the control and model groups were injected with 500 UL of saline via the tail vein, and rats in the nalbuphine group were injected with nalbuphine solution at a dose of 1 mg/100 g in the tail vein. Three minutes later, the control group rats were accepted with an intraperitoneal injection of saline at 1 ml/100 g body weight, and rats in the model and nalbuphine groups were injected intraperitoneally with 1% acetic acid at 1 ml/100 g body weight.

### Behavioral measurements

The writhing test was adapted from the description made by Schmauss and colleagues [35]. Briefly, a positive reaction was characterized by: dorsal-claw flexion to the left or right, body extension and abdomen flat, a contraction in the abdomen followed by extension of the hind limbs. The number of positive responses was recorded every 5 min for 30 min and recorded as the writhing times.

Paw withdrawal threshold (PWT) was measured using a set of Von Frey monofilaments (VFMs; Aesthesio®; DanMic Global, LLC, American). The target force of VFMs was 0.6, 1, 2, 4, 6, 10, 15, and 26 g using the up-down method described by S.R. Chaplan, and Bradman [36, 37]. Rats were placed in a plastic cover on a wire mesh floor (5 mm × 5 mm) and allowed to habituate for 30 min before testing. Starting from a target force of 4 g, a series of VFMs was tested vertical to the middle (not the pad) of the right lateral plantar surface of the paw for 7 s per VFM. A low-level VFM was used if the agile withdrawal of a paw was observed; otherwise, a high-level VFM was applied. When the first positive reaction was followed by a negative reaction or vice versa, four additional stimuli were administered. Each rat received only one treatment, and researchers were blinded to the treatment applied. Experimental data were converted in the way described by Chaplan and colleagues [38].

### Enzyme-linked immunosorbent assay (ELISA)

TNF-α, IL-1β, IL-2, IL-6 in the plasma of rats were measured in duplicates usingEnzyme-linked immunosorbent assay according to manufacturer instructions. Plasma was extracted from the supernatant of whole blood collected from rat hearts after centrifugation for 15 min at 2.415(× g) at 4 °C, and stored at − 80 °C. 1:5 dilution of plasma was used for the quantification of all cytokines. Each ELISA kit 96 T (Sinovac Biotech. Shanghai, China) consists of a prepared antibody-coated microtiter plate, washing solution, HRP-conjugated reagent, Standards, Standards solution, Samples solution, TMB substrate solution and Stop solution. Samples and standards were loaded and incubated for 45 mins at RT. Streptavidin-horseradish peroxidase conjugate was treated and incubated for 30 mins. TMB substrate solution was treated for 10 mins, and then Stop solution was added. Washing each well with washing solution was performed between every step. Optical density (O.D.) was measured at 450 nm using EnSpire® Multimode Plate Reader (PerkinElme, USA). Detection range of standards for IL-1β was 1 ng/L to 40 ng/L; IL-6 was 3 pg/ml to 120 pg/ml; TNF-α was 10 ng/L to 360 ng/L; and IL-2 was 100 ng/L to 1800 ng/L.

### Western blotting

Total protein in the spinal cord (L3–5) was analyzed by western blot. Briefly, the spinal cord was homogenized in RIPA lysis buffer containing phenylmethylsulfonyl fluoride and phosphorylase inhibitor cocktail A (50×) according to specifications using a Polytron™ homogenizer five times, 10s each, 1 min apart. After the protein had been lysed fully on ice for 20 min, the protein was centrifugated for 5 min at 12000(×g) at 4 °C and calculated the concentration using a bicinchoninic acid kit (P0012s, Beyotime) following instructions. After mixing with sodium dodecyl sulfate-polyacrylamide gel electrophoresis (SDS-PAGE) sample loading buffer (5×; P0015, Beyotime), protein samples were degenerated at 100 °C for 10 min by a Mixer-heat/Cool mixer (Thermo Scientific, TCS10, Waltham, MA, USA) and stored at − 80 °C. Proteins were separated by SDS-PAGE and then transferred to polyvinylidene fluoride (PVDF) membranes (Millipore, Bedford, MA, USA) with an aperture of 0.45 mm. PVDF membranes were blocked with 5% non-fat dry milk containing 0.05% Tween 20-TBS for 2 hours at room temperature and then incubated with primary antibodies against the anti-GAPDH(1:1000), anti-TNF-α (1:200), anti-IL-6 (1:1000), anti-IL-1β (1:1000), anti-IκBα (1:1000), anti-phosphorylated (p)-NF-kB p65 (ser536) (1:1000) overnight at 4 °C. Then, immunoblots were incubated with the horseradish peroxidase (HRP) conjugated secondary antibody appropriated to its primary antibody for 2 hours at room temperature and observed by an ECL detection kit (P0018AS, Beyotime). Imaging software (ChemiDoc™ Touch; Bio-Rad Laboratories, Hercules, CA, USA) was used to scan each immunoblot and record its image.

### Real-time reverse transcription-quantitative polymerase chain reaction (RT-qPCR)

Spinal cord (L3–5) separated from rats in different groups under isoflurane anesthesia and collected in a frozen storage tube without RNase or DNase (1.8 mL; Corning). This tissue was stored in liquid nitrogen until further processing. RNA was isolated using TRIzol® Reagent (R0016, Beyotime) and purified with DNase I according to the instructions. Complementary-DNA was synthesized using the Reverse Transcription cDNA Archive kit (Bio-Rad, T100, USA). Expression of IL-1β, IL-6, NF-κB p65, and TNF-α was measured by the CFX96™ Real-Time System (Bio-Rad, USA) and TaqMan™ Gene Expression kit (RR047A, TaKaRa Biotechnology). A two-step PCR protocol was used to amplify and each step was based on instructions provided by the manufacturers. Data were collected and the quantification (Cq) was utilized to calculate the gene expression of IL-1β, IL-6, NF-κB p65, and TNF-α in all groups using GAPDH expression to normalize (2 − ΔΔCT method). Primer sequences are shown in Table 1.

**Table 1:** Primer sequences used for RT-qPCR

Gene name	Sense primer (5′-3′)	Anti-sense primer (5′-3′)
IL-1β	TCCCAAACAATACCCAAAGAAG	ACTATGTCCCGACCATTGCTG
IL-6	GACAAAGCCAGAGTCATTCAGAG	GGATGGTCTTGGTCCTTAGCC
NF-KB p65	ACCTGGAGCAAGCCATTAGC	CCGCATTCAAGTCATAGTCCC
TNF-α	CTTCTCATTCCTGCTCGTGG	CCGCTTGGTGGTTTGCTAC

### Immunohistochemical (IHC) analyses

IHC experiment was undertaken following behavioral experiments. Under isoflurane anesthesia, the spinal cord was harvested after pre-fixation performed by injecting 100 mL of 0.9% saline and 200 mL of 4% paraformaldehyde into the heart. After fixation, tissue was placed in 4% paraformaldehyde for 72 hours. Then, specimens were dehydrated, embedded in paraffin, and sliced. Slices were dewaxed, hydrated, antigenic repair, blocked, and then incubated overnight at 4 °C with primary antibody. Then, they were incubated for 50 min at room temperature with HRP conjugated secondary antibody followed by DAB colored and microscopic examination (Olympus, SLIDEVIEW™ VS200, Tokyo, Japan). The primary antibodies for Anti-TNF-α (1:100) and anti-p-NF-kB p65 (ser536) (1:100) were applied.

### Statistical analyses

All data were expressed as mean ± standard error of the mean (SEM) and analyzed using SPSS26.0 software. Data were subjected to one-way ANOVA, nonparametric tests, or two-tailed Student’s t-test according to the results of testing for normality and homogeneity of variance. The post hoc test (LSD(L) or Bonferroni(B)) was conducted to compare multiple groups only if ANOVA yielded a significant main effect. *P* < 0.05 was considered significant. Graphs were made by GraphPad Prism 8 and grouped by AI or PS.

## Results

### Nalbuphine intervention made behaviors of acute visceral pain model rats changed

**Fig. 1:**
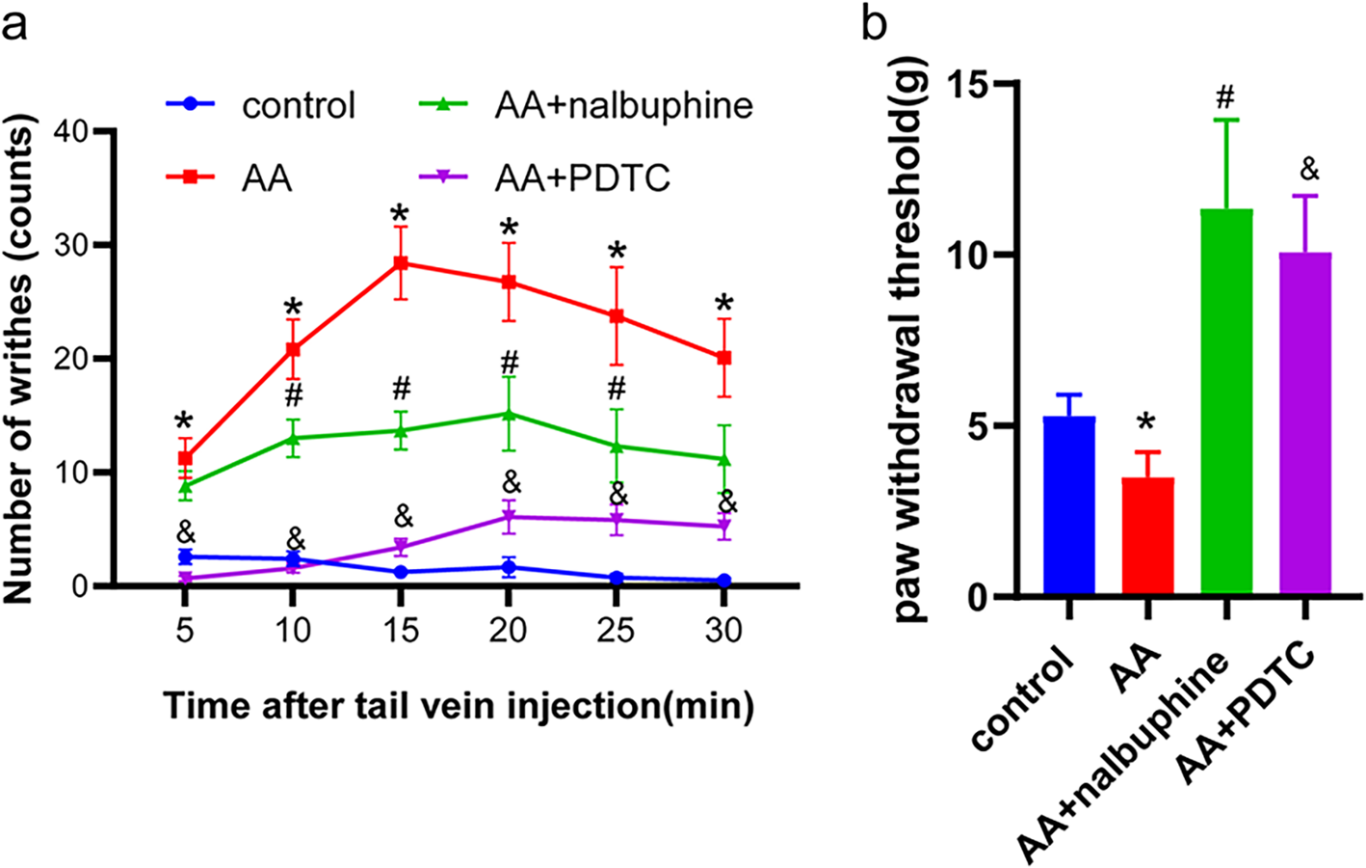
The effects of acetic acid, nalbuphine, and PDTC on the writhing test and paw withdrawal threshold (PWT). **a** The number of writhes for 30 minutes in each group. **b** Mechanical paw withdraw threshold in each group (data were the mean ± SEM, *n* = 12). *p < 0.05, AA vs control; ^#^*p* < 0.05, AA+nalbuphine vs AA, ^&^*p* < 0.05; AA+PDTC vs AA

To assess the pain response, writhing numbers and PWT were measured respectively. The writhing test showed numbers of writhes in the AA group and AA+nalbuphine group were higher than that in the control group (*p* < 0.05), and the analgesic effect of nalbuphine (10 μg/kg) in rats led to a significantly diminished number of writhing compared with that in the AA group (model group), started at 10 minutes (Fig.1a) (*p* < 0.05). In the mechanical paw withdraw threshold test, the PWT was decreased significantly in the AA group (3.50 ± 0.73 g) compared with that in the control group (5.29 ± 0.61 g), whereas it was increased in the AA+ nalbuphine (11.35 ± 2.59 g) and AA+ PDTC group (10.08 ± 1.63 g) (Fig. 1b) (*p* < 0.05). The results showed that nalbuphine and NF-κB inhibitor PDTC both exerted good analgesic effects in visceral pain.

### Nalbuphine suppressed the levels of IL-1β, IL-2, IL-6, and TNF-α in acute visceral pain rats

**Fig. 2:**
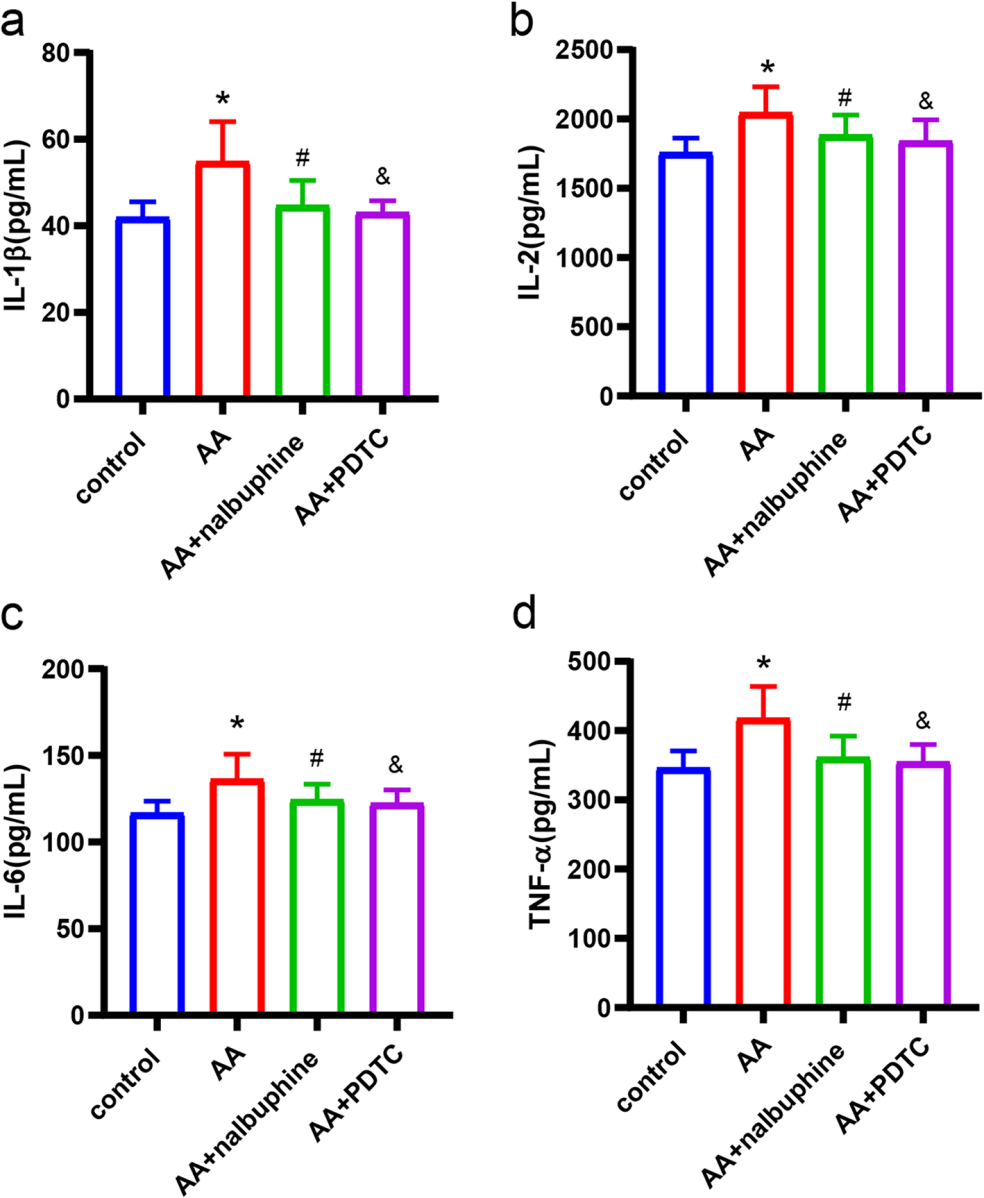
Results of plasma levels of several inflammatory factors. The expressions of IL-1β, IL-2, IL-6, and TNF-α were compared between groups (data were the mean ± SEM, *n* = 8). *p < 0.05, AA vs control; ^#^*p* < 0.05, AA+nalbuphine vs AA, ^&^*p* < 0.05; AA+PDTC vs AA.

ELISA results showed that AA-treated rats secreted higher levels of IL-1β, IL-2, IL-6, and TNF-α than control rats in plasma (Fig. 2a-d), whereas nalbuphine inhibited AA-stimulated increase in the above inflammatory factor levels. These results fully proved the anti-inflammatory effect of nalbuphine. PDTC as an inhibitor of NF-κB also played an anti-inflammatory role in acute visceral pain. The levels of IL-1β, IL-2, IL-6, and TNF-α in the PDTC group were significantly decreased than in the AA group.

### Nalbuphine modulated p-NF-κB p65 and IκBα in the spinal cord of visceral pain rats

**Fig. 3:**
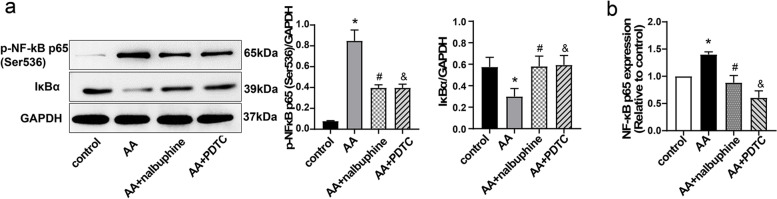
Levels of IκBα and p-NF-κB p65 (Ser536) in four groups. **a** The protein levels of IκBα and p-NF-κB p65 (Ser536) in the spinal cord were detected via WB. GAPDH was used as a loading control. **b** The mRNA expression of NF-κB p65 in the spinal was detected via RT-qPCR. (Data are the mean ± SEM, *n* = 4). *p < 0.05, AA vs control; ^#^*p* < 0.05, AA+nalbuphine vs AA, ^&^*p* < 0.05; AA+PDTC vs AA

NF-κB p65 usually binds to inhibitory IκBα proteins and remains silent in the cells. When phosphorylated IκBα becomes degraded, NF-κB p65 is released and translocated into the nucleus and finally leads to regulation of gene transcription [39, 40]. So, we measured both the levels of p-NF-κB p65 and IκBα using western blotting and RT-qPCR. The results showed that AA-treated rats decreased the level of IκBα and increased the protein expression of p-NF-κB p65 compared with control group rats (Fig. 3a). In addition, Fig.3b showed that nalbuphine and PDTC also inhibited an AA-stimulated increase in the mRNA level of NF-κB p65. Therefore, our study indicated that nalbuphine inhibited inflammation by down-regulating the NF-κB pathway in the spinal cord.

### IL-1β, TNF-α, and IL-6 were down-regulated by nalbuphine and PDTC in the spinal cord

**Fig. 4:**
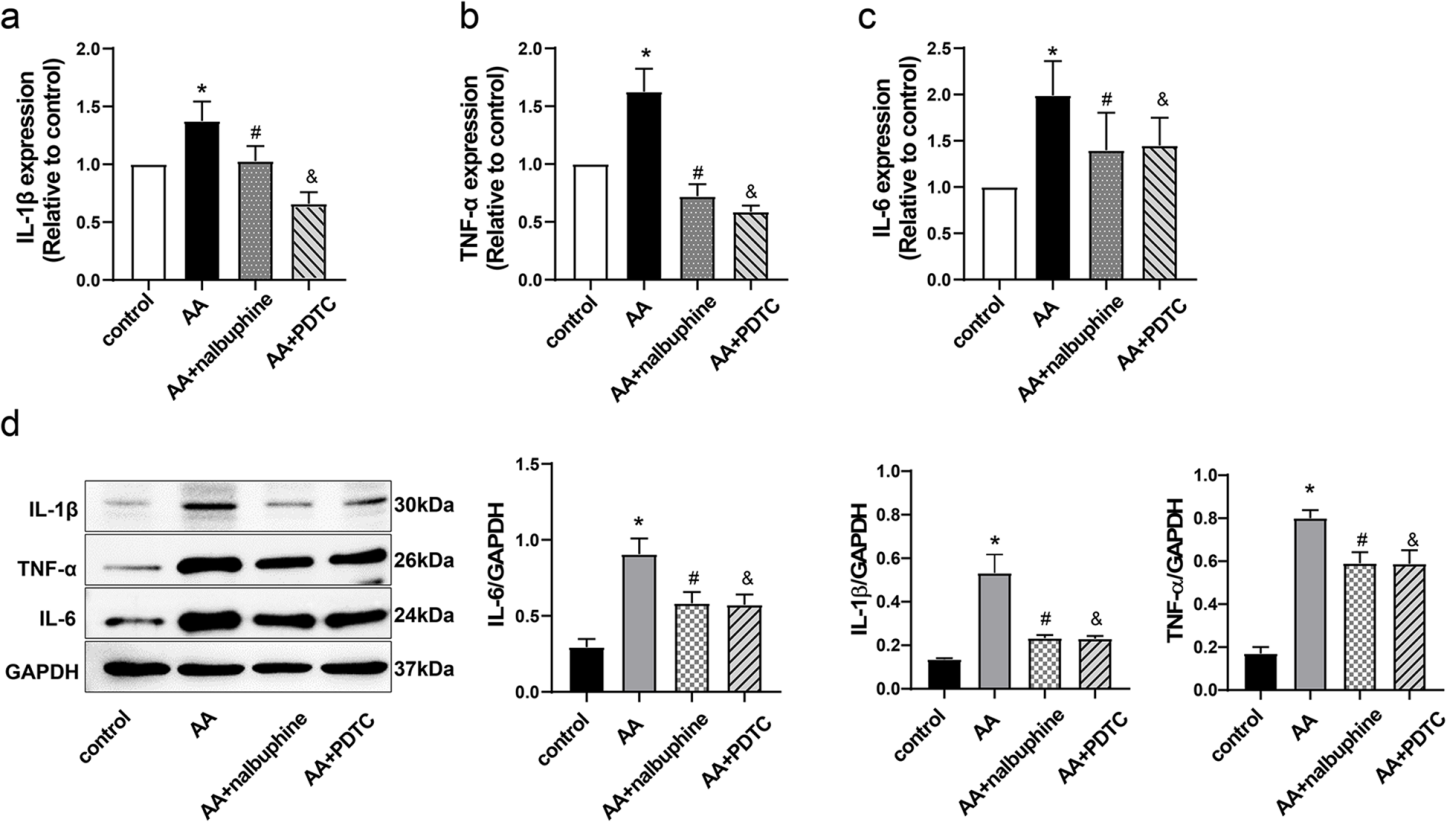
The mRNA and protein levels of IL-1β, TNF-α, and IL-6 in the lumbar spinal cord of rats. **a-c** The mRNA expressions of IL-1β, TNF-α, and IL-6 in the spinal cord were detected via RT-qPCR. **d** The protein levels of IL-1β, TNF-α, and IL-6 in the spinal cord were detected via western blotting. GAPDH was used as a loading control. (Data were the mean ± SEM, *n* = 4). *p < 0.05, AA vs control; ^#^*p* < 0.05, AA+nalbuphine vs AA, ^&^*p* < 0.05; AA+PDTC vs AA

To ascertain whether the inflammatory response in the spinal cord was increased during nalbuphine and PDTC treatment, we measured the expressions of IL-1β, TNF-α, and IL-6 in the lumbar spinal cord. Western blotting and RT-qPCR results suggested that acetic acid upregulated the proteins of IL-1β, TNF-α, IL-6, and their mRNA expressions. Nalbuphine treatment decreased the mRNA and protein levels of IL-1β, TNF-α, and IL-6 compared with rats in the AA group rats (Fig. 4a-d). This indicated that nalbuphine reduced the expression of inflammatory factors not only in the plasma but also in the spinal cord. The NF-κB inhibitor PDTC treatment also reduced the levels of IL-1β, IL-6, and TNF-α in the spinal cord compared to the AA group to exert anti-inflammatory effects.

### Nalbuphine decreased p-NF-κB and TNF-α protein levels in the white matter of the spinal cord

**Fig. 5:**
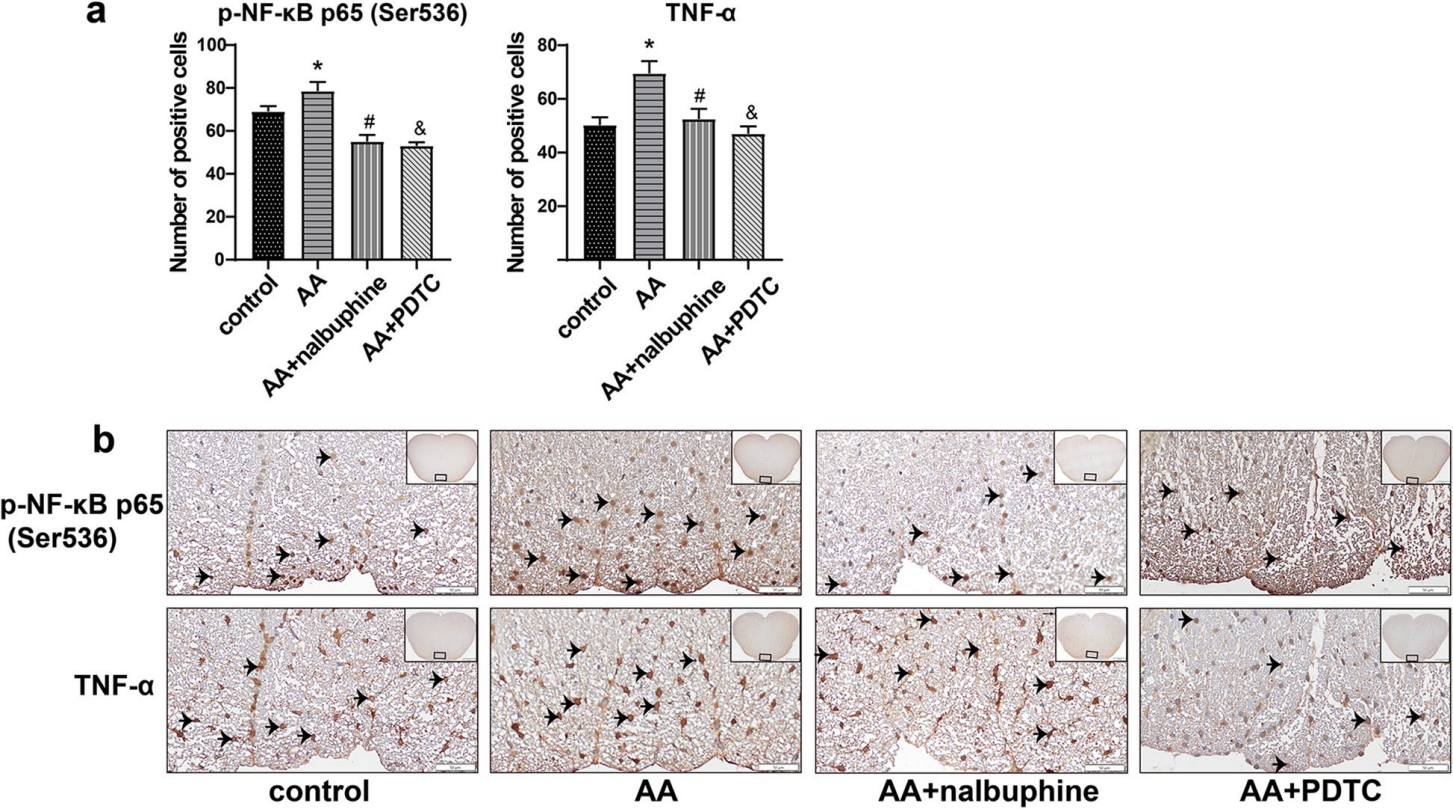
The expressions of p-NF-κB and TNF-α in the spinal were observed by IHC. **a** The number of positive cells at representative locations. **b** Representative images of p-NF-κB and TNF-α after immunohistochemical staining (SLIDEVIEW VS200; × 20, × 200). (Data are the mean ± SEM, *n* = 4). *p < 0.05, AA vs control; ^#^*p* < 0.05, AA+nalbuphine vs AA, ^&^*p* < 0.05; AA+PDTC vs AA

In our study, IHC analyses revealed that acetic acid enhanced p-NF-κB and TNF-α expression in the AA group, whereas nalbuphine could reverse this change. The result of IHC analyses consisted of western blot and RT-qPCR. So, we further confirmed that nalbuphine exerted its anti-inflammatory effect through down-regulating NF-κB. In addition, we found p-NF-κB and TNF-α mainly distributed in the white matter of the spinal cord in all group rats. (Fig. 5).

## Discussion

Visceral pain is a common clinical problem, it can be caused by chemical or physical damage, as well as by psychological, microbial, and endocrine factors, yet its mechanism is not fully clear [41–43]. The development of visceral pain is regulated by the spinal cord and higher centers of the brain. Visceral pain tends to company physical discomfort and special positions like knee flexion position in humans. Similarly, the number of writhes in a certain period reflects the degree of pain in rats and is often used in pain-model experiments [44–46]. Acute visceral pain like acute appendicitis tends to lead to superficial or deep hyperalgesia or allodynia [47]. In Liu’s research, the PWT will be reduced in rats when there is central sensitization of the spinal cord [48]. In our research, the analgesic drug nalbuphine significantly decreased the writhing numbers of rats caused by acetic acid and increased the PWT. It suggested that the central sensitization of rats is extremely obvious and rapid in the model of visceral pain induced by acetic acid, while nalbuphine could significantly inhibit this change. These experimental results are consistent with the data in other studies [44, 45].

TNF-α, IL-6, IL-1β, and IL-2 are involved in many inflammatory visceral pain diseases like visceral pain after colitis, surgery, and endometriosis [40, 41, 49]. Nalbuphine is applied in some clinical researches to play valid anti-inflammatory and analgesic effects [42, 43]. Besides these clinical studies, in ML’s study, expressions of pro-inflammatory cytokines (IL-6, IL-1β, and TNF-α) in the spinal cord were increased in visceral pain rats and inhibited by dexmedetomidine [44]. These studies indicate that inflammatory factors are widely expressed in visceral pain diseases. In our study, TNF-α, IL-6, IL-1β, and IL-2 significantly enhanced in rats in the visceral pain model induced by acetic acid, and nalbuphine decreased above inflammation factors not only in plasma but also in the spinal cord. It reveals that nalbuphine exerts its anti-inflammatory and analgesic effects through neurohumoral regulation in the central spinal cord system. These results were consistent with our clinic research before, nalbuphine inhibited inflammation after orthognathic surgery [32].

NF-κB participates in the regulation of cell growth, proliferation, inflammation, immunity, energy metabolism, tumor metastasis, and other aspects in organs [16, 50–56]. Besides, in YZ Li’s study, spinal NF-κB upregulation contributes to hyperalgesia in a rat model of advanced osteoarthritis [57]. Combined with the values of PWT in this study, it was hypothesized that the central sensitization of visceral pain caused by acetic acid was also associated with spinal NF-κB. Usually, NF-κB couples with IκBα in an inactive state in cells, and when the synthesis of IκBa is inhibited or decomposed, NF-κB activation increases [58]. IκBα as a key inhibitory protein of NF-κB inhibits the activation of NF-κB (p65) and then reduces expression of downstream pro-inflammatory factors like TNF-α [59, 60]. In the present study, visceral pain caused by acetic acid (i.p) could increase the expression of NF-κB (p65) and TNF-α, whereas decreased the level of IκBα in the spinal cord, especially in the macrophage/microglia at white matter of the spinal cord. The white matter is composed of oligodendrocyte precursor cells (OPCs), oligodendrocytes, astrocytes, and microglia [61]. Macrophage/microglia are extensively involved in the process of inflammatory response in the central nervous system [62, 63]. Our results also suggested that spinal macrophage/microglia are also involved in the development of inflammatory visceral pain. Blocking the activation of NF-κB with NF-κB inhibitor (PDTC) remarkably suppressed the AA-induced expression of TNF-α. So, we concluded that increased NF-κB expression in the spinal white matter is a key central regulating mechanism in visceral pain. Analgesic drug nalbuphine significantly inhibited NF-κB (p65) and TNF-α and increased the IκBα protein expression in the spinal cord, also mRNA and IHC tests showed consistent results in NF-κB (p65) and TNF-α. The results suggested that nalbuphine, as a k-receptor agonist exerted its anti-inflammatory effects by inhibiting the NF-κB pathway.

So far, our research has explored from clinical phenomenon to mechanism in the spinal cord of rats and finally found that nalbuphine can exert anti-inflammatory and analgesic effects on the inflammatory visceral pain caused by acetic acid through downregulating NF-κB in the spinal cord.

## Conclusion

In conclusion, nalbuphine could inhibit the strong response of acute inflammatory visceral pain by inhibiting NF-κB (p65) activation to reduce the release of downstream pro-inflammatory cytokines.

## Supplementary Information

**Additional file 1.**

## Data Availability

All data generated or analyzed during this study are included in this published article.

## Abbreviations

ELISA: Enzyme-linked immunosorbent assay
TNF: Tumor necrosis factor
IL: Interleukin
p-NF-κB p65: Phosphorylated nuclear factor-kappa B
IHC: Immunohistochemical
AA: Acetic acid
VFM: Von Frey monofilament
SDS PAGE: Sodium dodecyl sulfate-polyacrylamide gel electrophoresis
PVDF: Polyvinylidene fluoride
RT-qPCR: Real-time reverse transcription-quantitative polymerase chain reaction
PWT: Paw withdrawal threshold
PDTC: Pyrrolidine dithiocarbamate
