# Supplementary material for: Nalbuphine alleviates inflammation by down-regulating NF-κB in an acute inflammatory visceral pain rat model
Source: BMC Pharmacol Toxicol. 2022 Jun 1;23:34. doi: 10.1186/s40360-022-00573-7 (PMC9158276; doi:10.1186/s40360-022-00573-7)
Supplement: Supplementary file 1 — Additional file 1. [file 40360_2022_573_MOESM1_ESM.pdf]

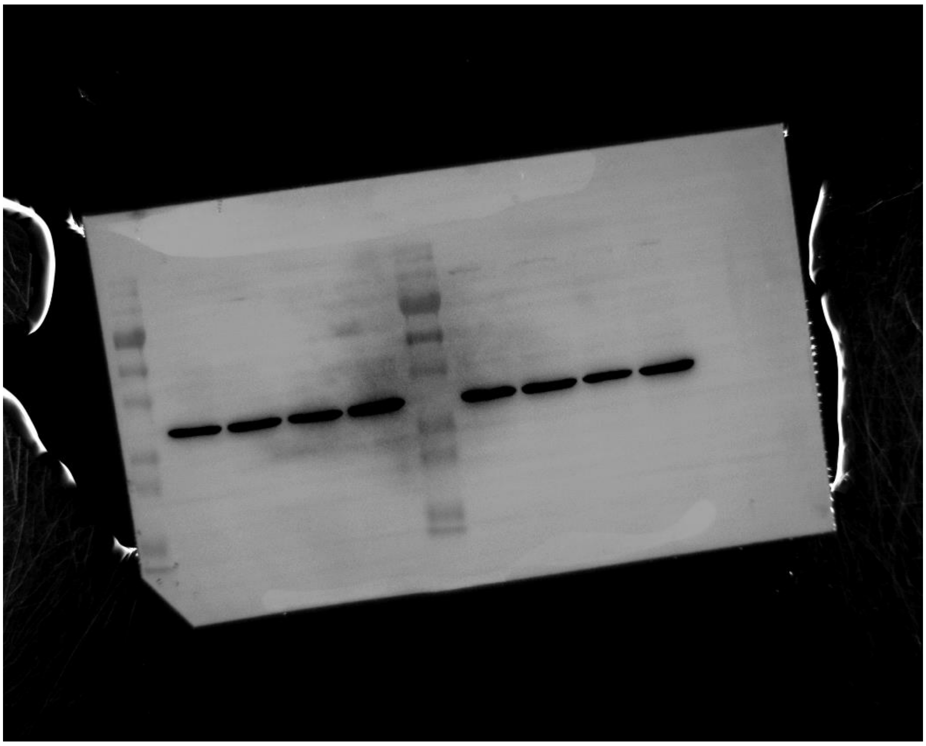

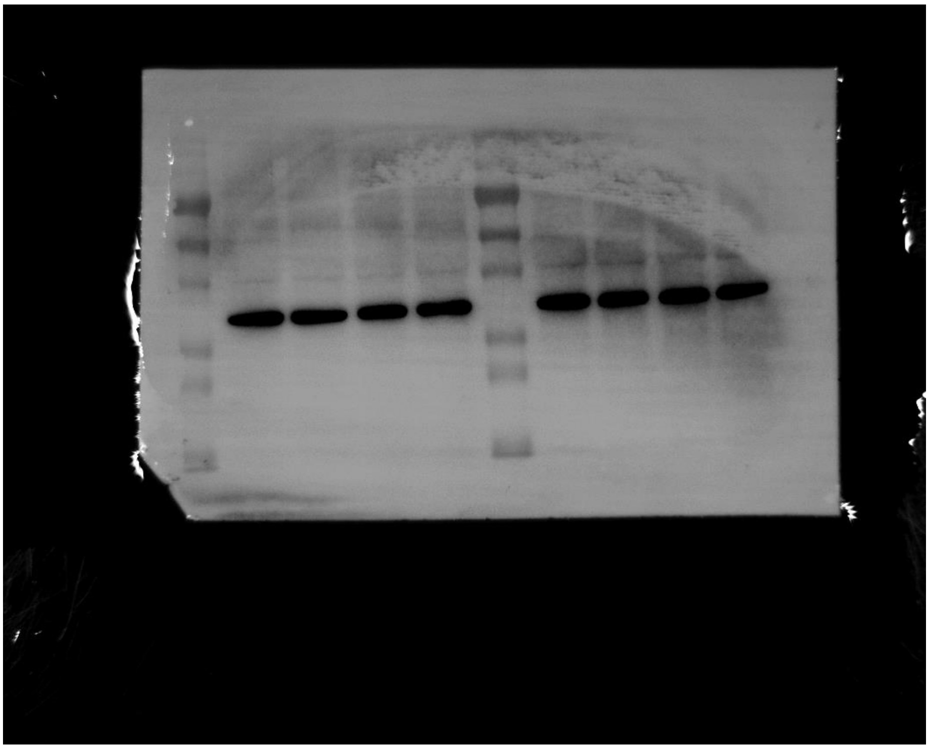

4 replicates of GAPDH

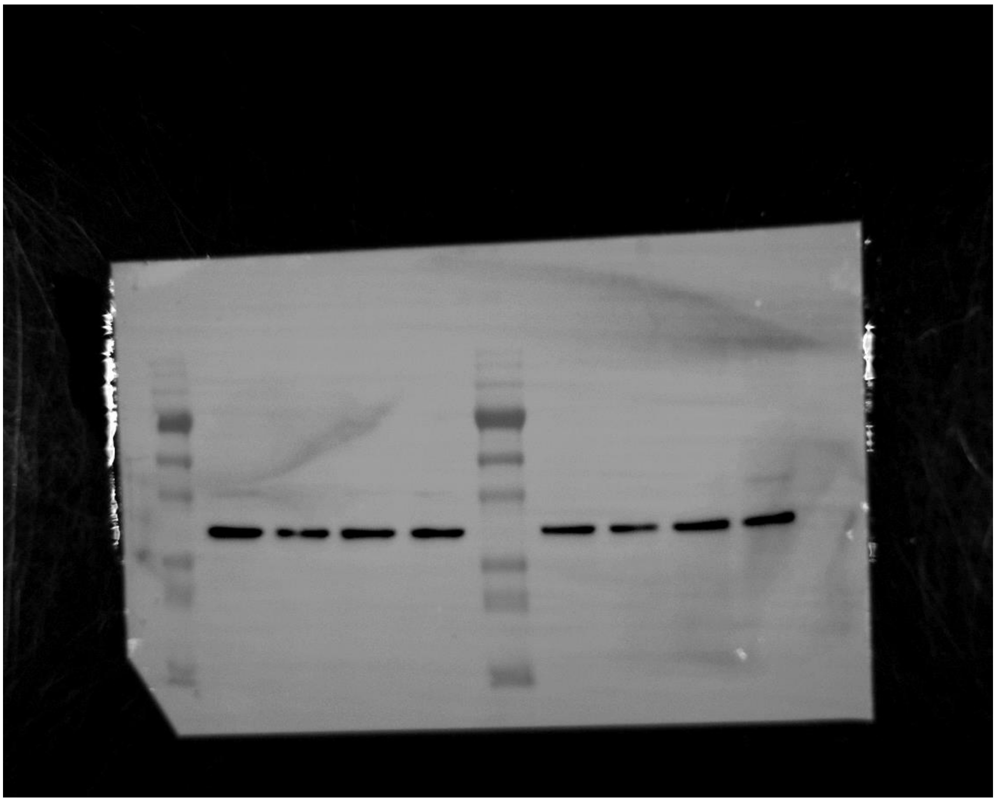

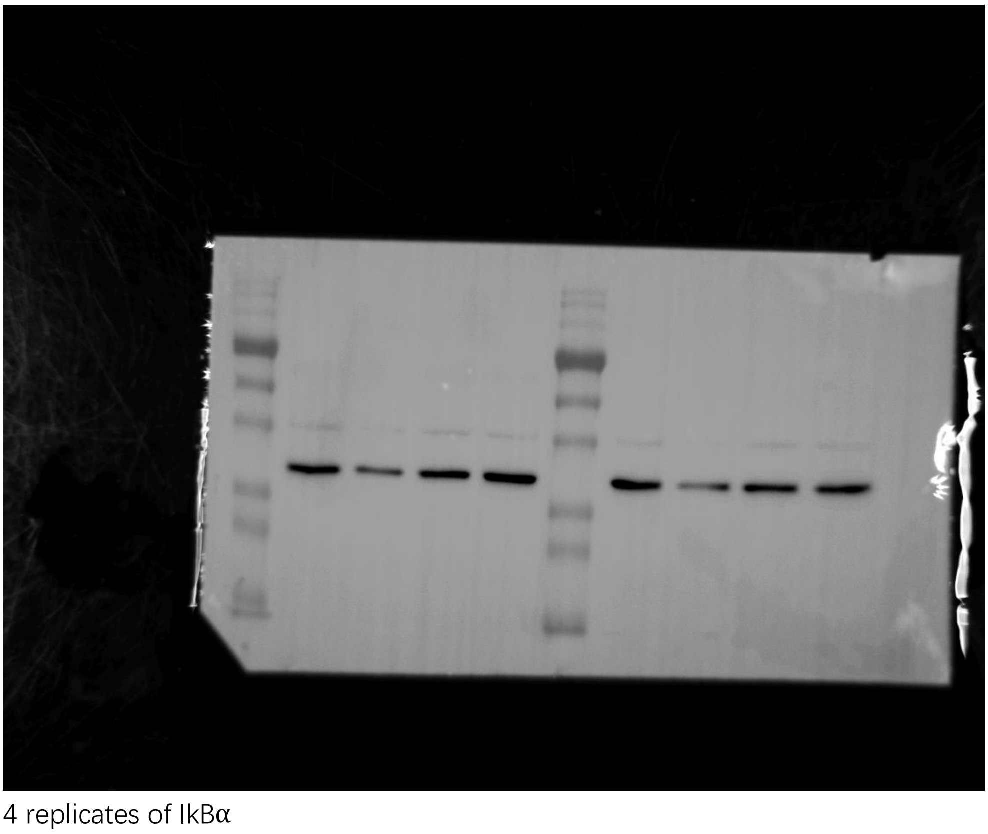

4 replicates of IkBα

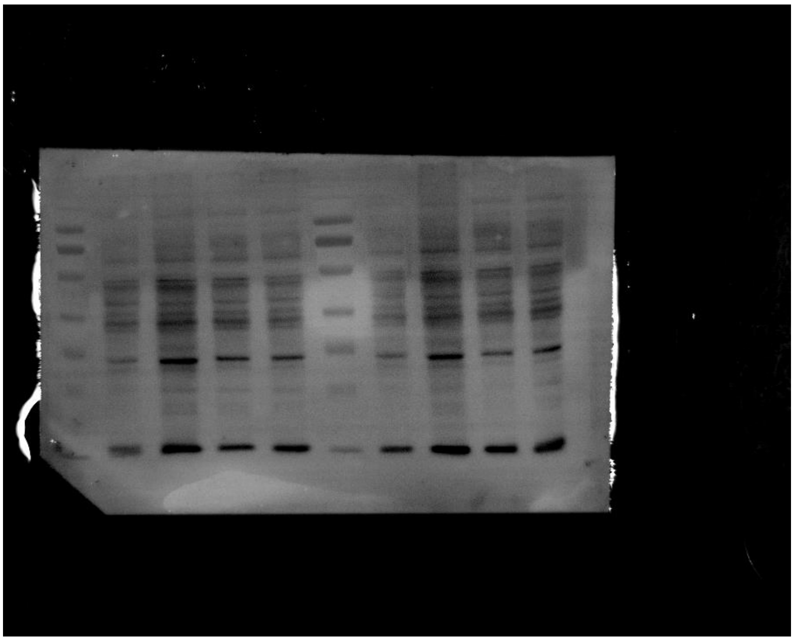

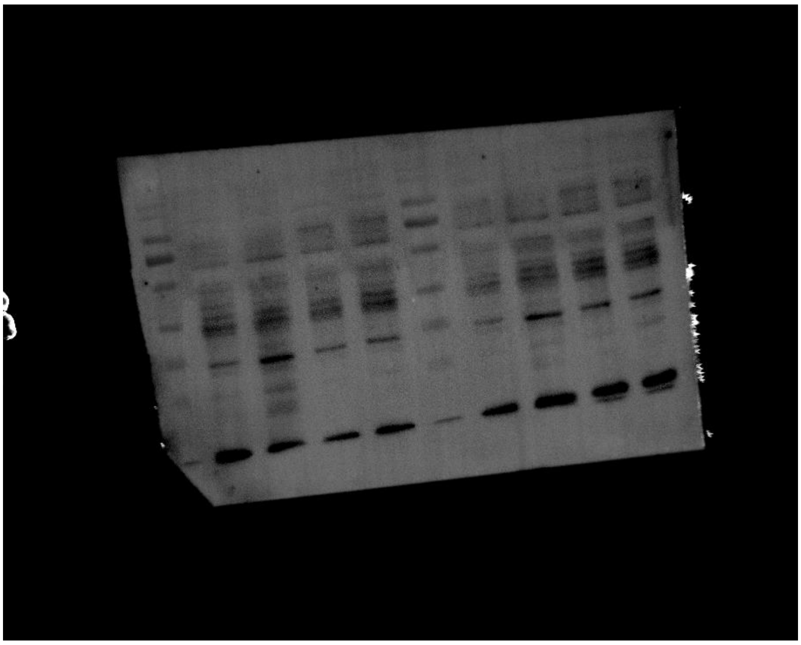

4 replicates of IL-1β

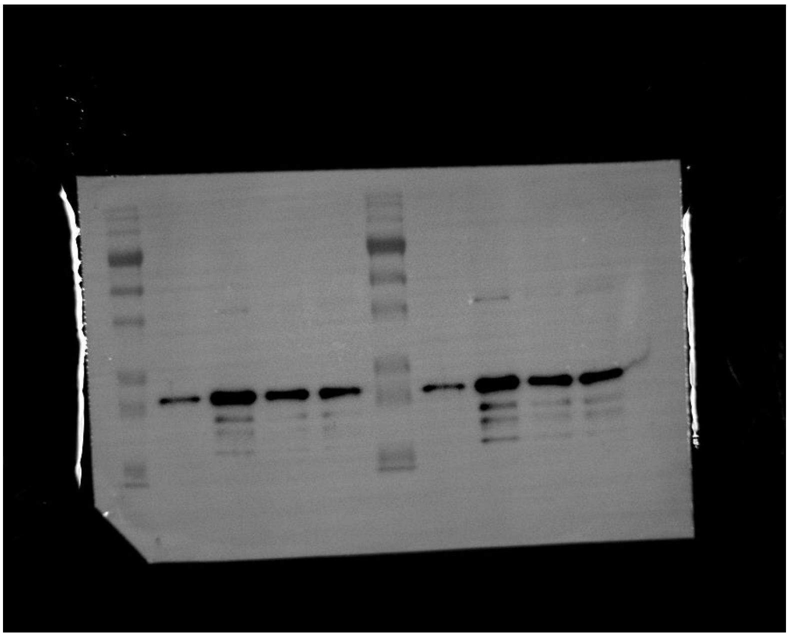

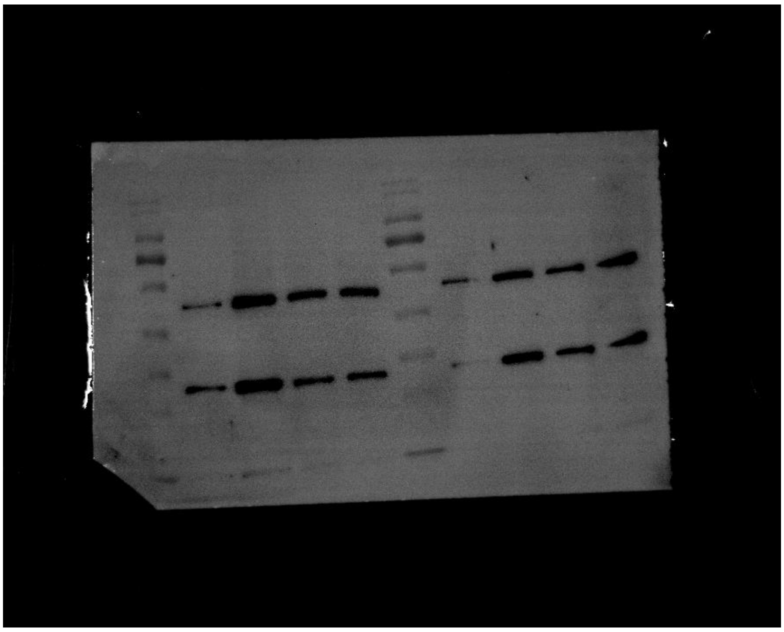

4 replicates of IL-6

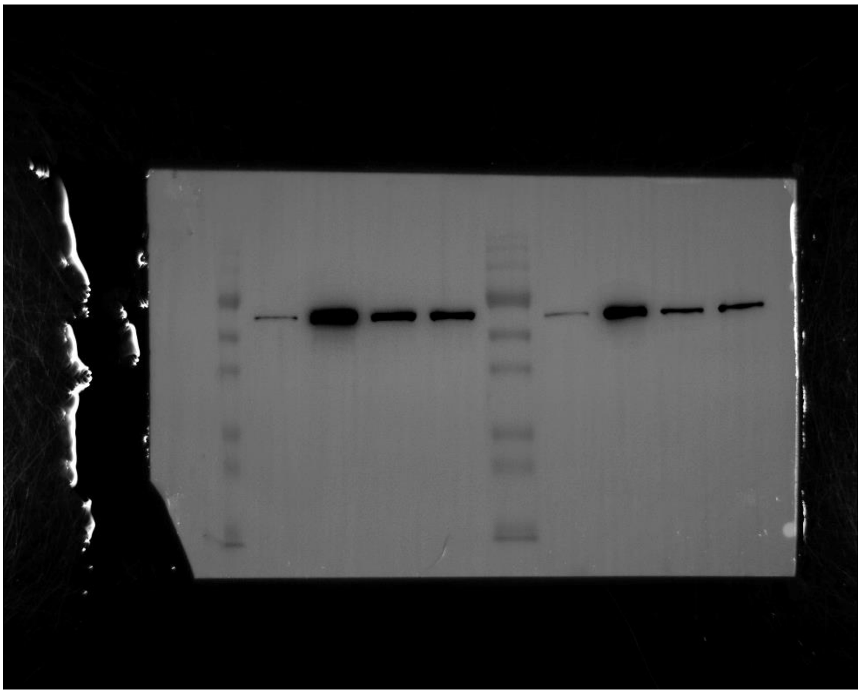

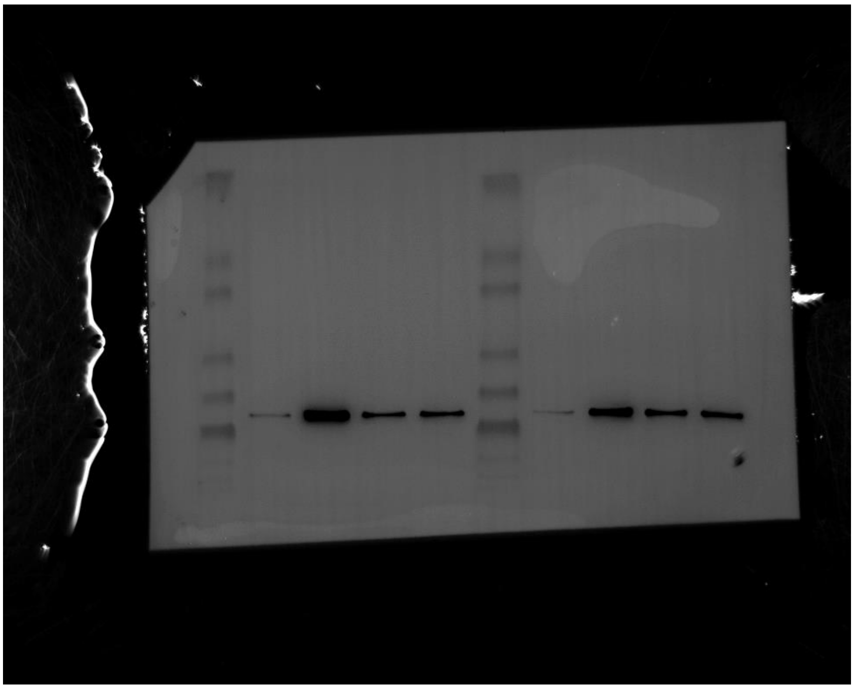

4 replicates of p-NF-κB

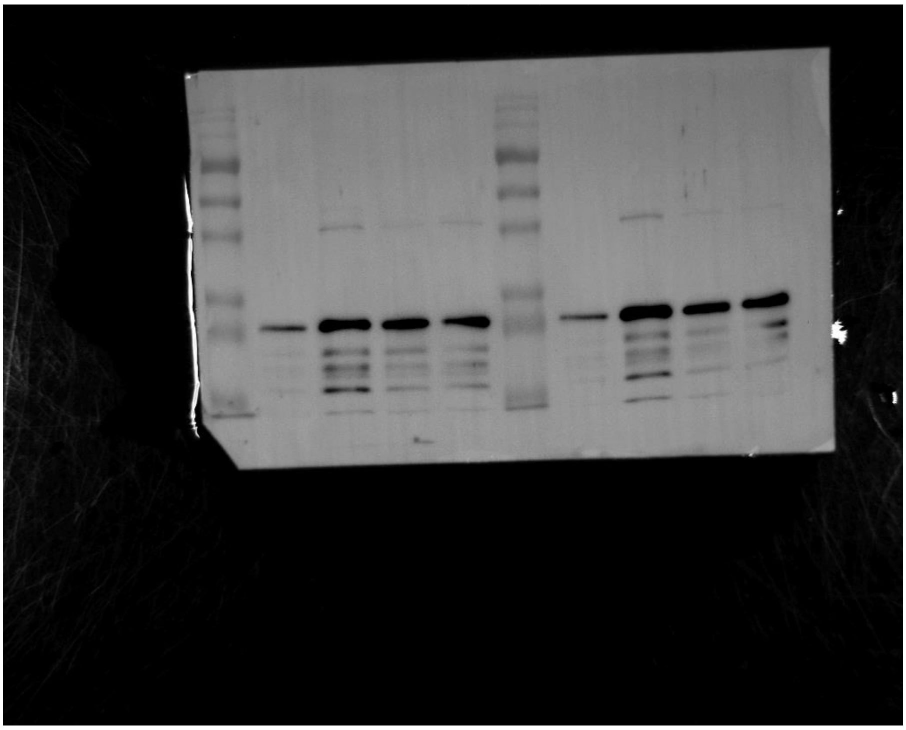

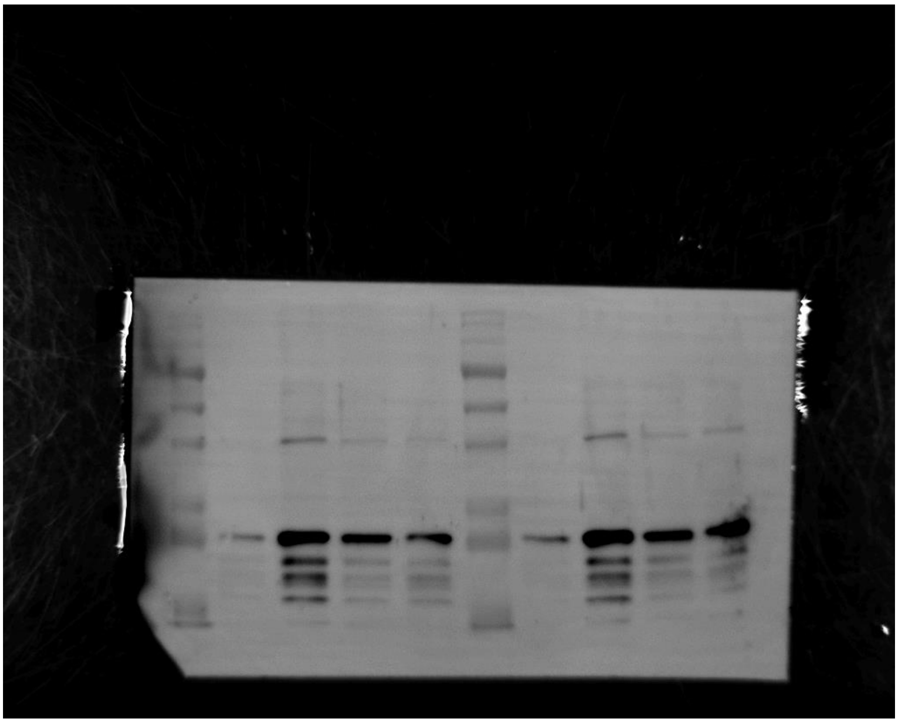

4 replicates of TNF-α
